# Gynaecologic oncology surgical cancellations in Zambia

**DOI:** 10.3332/ecancer.2023.1617

**Published:** 2023-11-02

**Authors:** Samson Chisele, Mulindi Mwanahamuntu, Paul Kamfwa, Mukatimui Kalima-Munalula, Swali Fundafunda, Kenneth Chanda, Maya M Hicks, Leeya F Pinder, Krista S Pfaendler, Groesbeck P Parham, Michael L Hicks

**Affiliations:** 1 Department of Obstetrics and Gynecology, University Teaching Hospital – Women and Newborn Hospital, Lusaka 10101, Zambia; 2 Cancer Diseases Hospital, Lusaka, 10101, Zambia; 3 Anne Arundel Medical Center, Department of Obstetrics and Gynecology, 2000 Medical Pkwy, Belcher Pavilion, Ste 309, Annapolis, MD 21401, USA; 4 University of Cincinnati College of Medicine, Ob/Gyn, Cincinnati, OH 45267, USA; 5 Department of Obstetrics and Gynecology, West Virginia University School of Medicine, 64 Medical Center Drive, Morgantown, WV 26506, USA; 6 Department of Obstetrics and Gynecology, University of North Carolina at Chapel Hill, 101 Manning Drive, Chapel Hill, NC 27514, USA; 7 St. Joseph Mercy Oakland Cancer Center, Michigan Cancer Institute, 44405 Woodward Ave, Suite 202, Pontiac, MI 48324, USA

**Keywords:** surgical cancellations in LMIC, gynaecologic surgical cancellations in LMIC, blood loss and surgical cancelations in gynaecologic oncology, lack of blood and surgical cancellations

## Abstract

**Introduction:**

Cancellations of elective surgery in low-and middle-income countries (LMIC) are common and a major hindrance for patients who are in need of surgical therapeutic modalities. This is especially important in the context of scaling up needed surgical interventions for gynaecological cancer care. There is a knowledge gap in the literature related to cancellation of gynaecologic oncology surgeries in LMIC, where there is enormous need for this specific cancer surgical capacity. We report in an observational descriptive fashion, our experience at the UTH/CDH in Lusaka, Zambia, on the causes of surgical cancellations in gynaecologic oncology.

**Methods:**

From January 1, 2021 through June 31, 2023, we retrospectively evaluated the surgical registry for gynaecologic oncology at the UTH/CDH in Lusaka, Zambia to assess the number and causes of surgical cancellations.

**Results:**

There were a total of 66 (16.96%) surgical cancellations out of 389 scheduled gynaecologic oncology cases. Lack of available blood and/or low haemoglobin was the most frequent cause of surgical cancellations, 27 cases (40.90%).

**Conclusion:**

We highlight in our series that the lack of blood, leading to surgical cancellations was the most frequent impediment related to performing scheduled gynaecologic oncology surgical procedures. As gynaecologic oncology services scale up in LMIC, given the radical nature of surgery and its association with blood loss, it is incumbent on the entire clinical ecosystem to address this issue and to develop mitigating strategies, specific to their respective resource setting.

## Introduction

Surgical cancellations for elective surgery in low- and middle-income countries (LMIC) are not uncommon. Reports have evaluated this problem on a broad perspective, inclusive of evaluating social determinants and infrastructural challenges while collectively looking across different surgical service lines [1–8]. These cancellations are a major hindrance for patients required to receive needed surgical care. This is especially important in the context of cancer care where it is predicted that only 5% of patients in LMIC will receive safe, affordable and timely surgery [9].

There appears to be a gap in the literature related to cancellation of surgery specific to the specialty of gynaecologic oncology. This is an important subject to investigate, especially in the context of the World Health Organization’s (WHO) 90/70/90 initiative (WHO November 17, 2020, Global Strategy) to reduce the burden of cervical cancer in LMIC. In sub-Saharan Africa cervical cancer is the leading cause of death among women and the second most common form of cancer [10]. Consistent with the WHO’s initiative, as screening increases in LMIC, it is estimated that up to 50% of these patients will be identified to have early stage, potentially curable disease [11]. For a cure to occur in comporting with the WHO management recommendations, these early-stage lesions require radical cancer surgery, a radical abdominal hysterectomy and pelvic lymphadenectomy. Therefore, identifying the causes of surgical annulment in this environment is extremely important.

At the University Teaching Hospital (UTH) and Cancer Diseases Hospital (CDH) in Lusaka, Zambia, we retrospectively evaluated all cancellations of surgical cases specific to the gynaecologic oncology service. We report here in an observational descriptive fashion our findings and suggest recommendations targeted for future improvements.

## Methods

From 1 January 2021 through 31 June 2023 we retrospectively evaluated the surgical registry for gynaecologic oncology at UTH and CDH in Lusaka, Zambia, to assess the number of cases resulting in surgical cancellations. All listed causes of cancellations were identified in order to determine the most prevalent causes of surgical cancellation.

## Results

There were a total of 66 (16.96%) surgical cancellations out of 389 scheduled cases for gynaecologic oncology surgeries (Table 1). Causes of surgical cancellation identified are as follows: 27 cases (40.90%) from low haemoglobin and lack of blood, 10 cases (15.15%) were no longer amenable to surgery, 10 cases (15.15%) were cancelled for other reasons not determined from available data sources, 7 cases (10.61%) from unavailable anaesthesiologists, 4 cases (6.06%) from severe uncontrolled hypertension, 3 cases (4.55%) from unavailability of surgeon, 2 cases (3.03 %) from deep vein thrombosis, 2 cases (3.03%) from failed intubation and 1 case (1.52%) from unavailable anaesthetic drugs (Table 2).

The specific cancers diagnosis and procedures that were cancelled, excluding the two cases of failed intubations, were as follows: 18 stage IB cervix cancers for radical hysterectomy with pelvic lymphadenectomy, 14 ovarian cancers for debulking/cytoreductive surgery, 12 endometrial cancer for total abdominal hysterectomy with pelvic and paraaortic lymph node sampling, 8 vulva cancers for radical vulvectomy with inguinal-femoral lymphadenectomy, 8 persistent high grade squamous Intraepithelial lesion of the cervix for simple hysterectomy, 5 large vulvar condylomas for simple vulvectomy and 1 vaginal cancer for wide radical excision (Table 3).

## Discussion

Across surgical service lines in LMIC operative cancellations of scheduled surgical cases range from 18% to 31% [1–8]. Most common causes of reported cancellations were surgeons or anaesthesiologist unavailability; lack of operating theatre time, equipment, ancillary surgical personnel and electricity; and improper surgical scheduling. These important infrastructural and social determinant barriers to surgery in LMIC have been well documented and it was not our intent to readdress these already reported issues. Furthermore, these reports elucidate a void in the literature with no mention of gynaecologic oncology surgery specifically, or link to how these surgical impediments impacts the management of a specific cancer disease entity endemic to LMIC identified by the WHO for eradication. Our study focused specifically on causes of surgical cancellations in gynaecologic oncology. As gynaecologic oncology surgical capacity increases in LMIC in an attempt to achieve the WHO’s initiative, it is important to know specific barriers that potentially will be faced related to surgical cancellations. To our knowledge to date this has not been reported in the literature.

In our series, we report a surgical cancellation rate of 16.96%. This number is slightly lower than the cited rates reported in the literature [1–8]. Although our number of cases is similar to other published reports (146 cases [1], 369 cases [7], 243 cases [8]), this lower rate may be a reflection associated with a specific single surgical oncological service analysis, the fact that we had no documented causes of cancellations related to social determinants such as lack of electricity and fewer cases being cancelled related to lack/absence of professional personnel (surgeon or anaesthesiologist), given that this centre is the only major referral centre for gynaecologic oncology in the entire country [1, 7, 8]

The most common identified reason for surgical cancellations was having a low haemoglobin and/or the lack of blood products (40.90%) being available on the date of scheduled surgery. Lack of blood products were not considered to be the most dominant cause of surgical cancellations in other reported studies; nor was there mention if blood loss was a common associated complication with a proposed oncological surgical procedure [1–8]. In the specialty of gynaecologic oncology this finding is of enormous importance. In LMIC cervical cancer is the second most common malignancy in women, incidence and mortality rates in sub-Saharan Africa are among the highest in the world, and is the leading cause of cancer deaths in women [10]. However, approximately 40%–50% of these patients have potentially preventable deaths secondary to having early stage disease that can be cured with a radical abdominal hysterectomy and pelvic lymphadenectomy [11]. This is why there is a major push to establish traditional and innovative ways to construct gynaecologic oncology surgical capacity in LMIC [12, 13]. However, it is vital to understand the challenges of radical pelvic surgery. A known common complication of this operation is intra-operative blood loss with the average amount being 1 L. This is much more common in LMIC where often these early stage cancer lesions will be much larger and surgery is often the only modality of therapeutic intervention in the absence of radiation and limited chemotherapy. Other gynaecologic oncological operations such as ovarian cancer tumour debulking, radical vulvectomy and inguinal-femoral lymphadectomy for large vulva cancer lesions, large vulva condyloma removals, radical vaginectomy and some nonradical hysterectomies are also associated with significant blood loss. These tumour groups represented the majority of the cancellations in our study. This consequential finding is important to document in the literature for the benefit of our institution and other equivalent LMIC centres in an effort to eliminate this principle impediment to surgical cancellations.

Reported causes of the lack of safe quality available blood in LMIC are attributed to many factors such as: transfusion-transmissible infections (HIV, Hepatitis B&C, malaria and syphilis), lack of benevolent donors, high demand on family donor-ship and lack of public awareness and political will [14, 15] Therefore, from a strategic perspective, while promoting for surgical capacity building in LMIC, it is important for stake holders and policy makers to simultaneously address and make improvements to ensure that these potentially preventable barriers for the availability of safe quality blood are curtailed. Solutions that have been put forth involve education at the local and governmental level related to developing better coordinated blood transfusion services, policies, regulation and legislation as well as expanding the pool of regular unpaid donors and rational use of blood to prevent unnecessary use. These goals and objective are very important and essential to the longevity of a country having safe available blood and the ability to build surgical capacity for gynaecologic malignancies. However, as these initiatives scale up, which we have implemented, in the immediate phases to prevent delays in oncological surgery, the dependency on family donors for current supplies will be essential. So, there will need to be implementation of a better focus on pre-operative assessment strategies to achieve this goal to prevent surgical cancellations.

Weaknesses of this study is that it is retrospective and depends on proper documentation in the cancellation logs of the surgical centre, which is subject to human variability and documentation errors. This is highlighted by the fact ten cases had no documented reason for the cause of delay. Another relative weaknesses is related to the overall number of scheduled surgical cases during the documented study period. This is secondary to the information being exclusively obtained from a single cancer surgical service line, gynaecologic oncology, that is the only referral centre in the country. Nevertheless, our number of surgical cases comports with other reported published series; and we were able to clearly show an important relevant contributing cause of surgical cancellations in gynaecologic oncology. Additionally, another contributing factor of reduced numbers in our series may be related to the COVID-19 pandemic in the 2021 timeframe.

## Conclusion

We highlight in our series that the lack of blood, leading to surgical cancellations was the most frequent impediment related to performing scheduled gynaecologic oncology surgical procedures. As gynaecologic oncology services scale up in LMIC, given the radical nature of surgery and its association with blood loss, it is incumbent on the entire clinical ecosystem to address this issue and to develop mitigating strategies, specific to their respective resource setting.

## Conflicts of interest

None.

## Funding

None.

## Figures and Tables

**Table 1.** Total number of cases and cancelations.

Number of planned surgical cases	Number of cancelled cases	Percentage
389	66	16.90

**Table 2.** Causes and percentage of surgical cancellation.

Cause of surgical cancellation	Number of cancellations	Percentage
No blood availability and/or low haemoglobin	27	40.91
Reason undetermined	10	15.15
Failed intubation	2	3.03
Deep venous thrombosis	2	3.03
Unavailable anaesthesiologist	7	10.61
Unavailable surgeon	3	4.55
No longer operable	10	15.15
Uncontrolled hypertension	4	4.17
Unavailable anaesthetic agents	1	6.06
Total	66	100

**Table 3.** Specific cancers diagnosis and procedures that were cancelled.

Cancer diagnosis	Operative procedure cancelled	Number
Cervix cancer	Radical hysterectomy with pelvic lymphadenectomy	18
Ovarian cancer	Ovarian cancers for debulking/cytoreductive surgery	14
Endometrial cancer	Total abdominal hysterectomy with pelvic and paraaortic lymph node sampling	12
Vulvar cancer	Radical vulvectomy with inguinal-femoral lymphadenectomy	8
Vulvar condyloma extensive	Simple vulvectomy	5
Cervical dysplasia	Simple hysterectomy	8
Vaginal cancer	Wide radical excision	1
Total		66
